# Supplementary material for: Spatial transcriptomics reveals niche-specific enrichment and vulnerabilities of radial glial stem-like cells in malignant gliomas
Source: Nat Commun. 2023 Feb 23;14:1028. doi: 10.1038/s41467-023-36707-6 (PMC9950149; doi:10.1038/s41467-023-36707-6)
Supplement: Supplementary file 1 — Supplementary Information [file 41467_2023_36707_MOESM1_ESM.pdf]

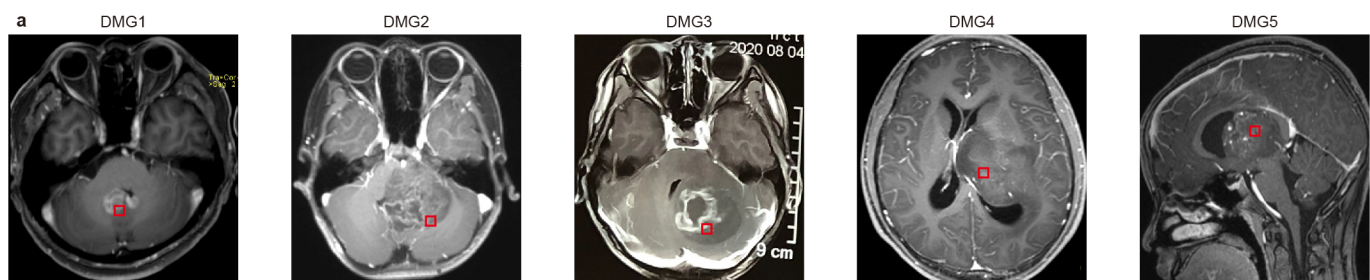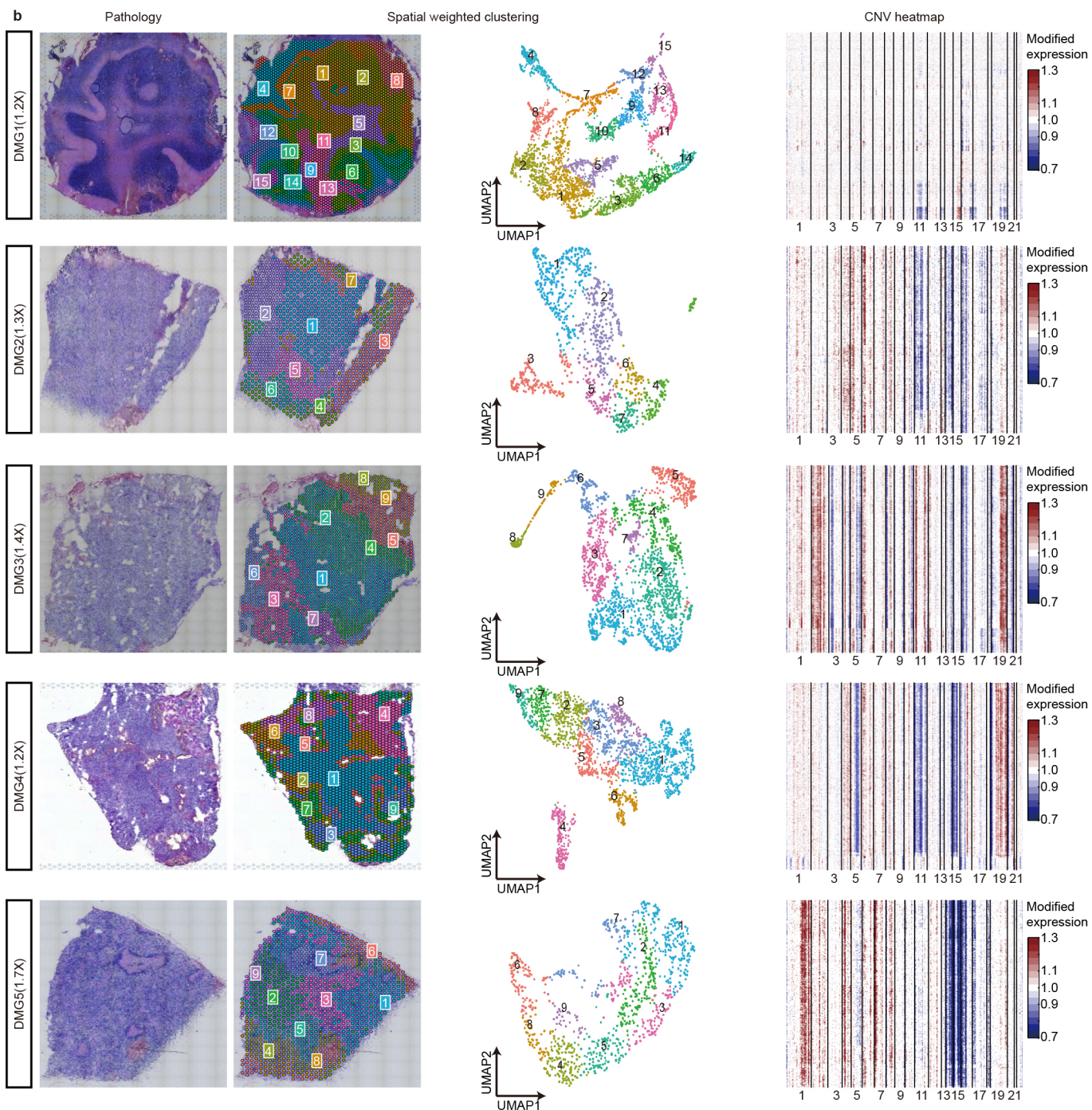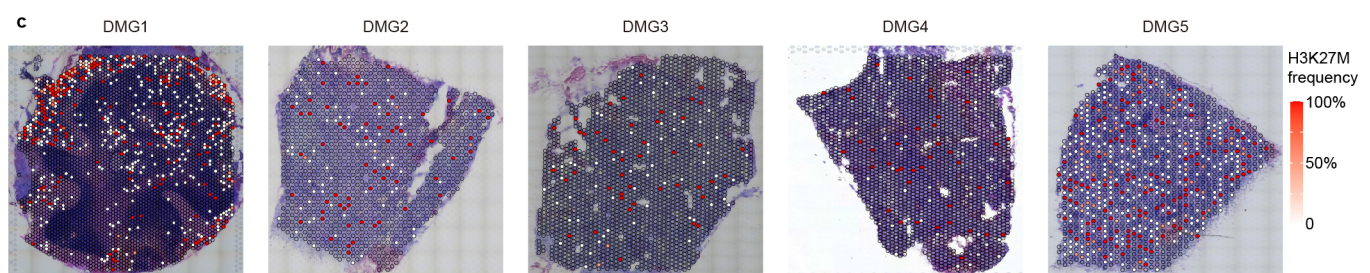

Supplementary Figure 1. MRI and spatial transcriptomic characterization of DMG samples.

- a) Contrast-enhanced T1-weighted MRI images of DMG1-5 show the tumor locations. The sampling sites are marked by red boxes.
- b) Left to right: H&E staining showing the histopathology, spatial/UMAP plots, and CNV heatmaps of DMG1-5. Spatial weighted clustering is based on BANKSY using default resolution parameters (0.8), visualized by Seurat. For CNV heatmaps, each row represents a spot and each column represents a chromosomal location. Red or blue represent chromosomal gain or loss, respectively.
- c) H3K27M mutational frequency in spots from DMG1-5. Colors from white to red represent the ratio of wildtype or H3K27M-mutant reads, respectively. Transparent spots contain no reads detected at the H3K27 locus.

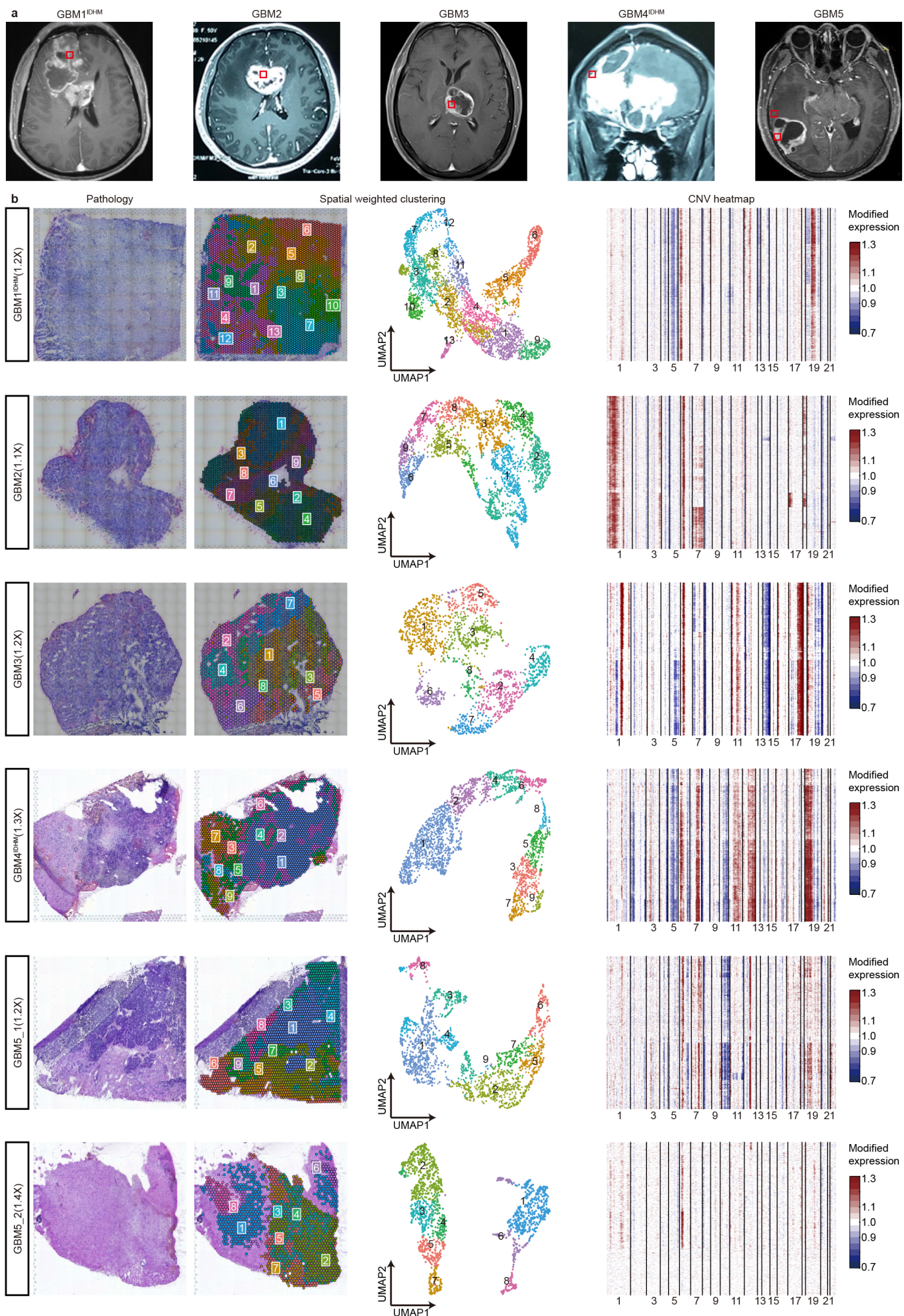

Supplementary Figure 2. MRI and spatial transcriptomic characterization of GBM samples.

a) Contrast-enhanced T1-weighted MRI images of GBM1-5 show the tumor locations, The sampling sites are marked by red boxes (GBM5_1 and GBM5_2 were sampled from the same patient in the cortex and peritumor area, respectively).

b) Left to right: H&E staining showing the histopathology, spatial/UMAP plots, and CNV heatmaps of GBM1-5. Spatial weighted clustering is based on BANKSY using default resolution parameters (0.8), visualized by Seurat. For CNV heatmaps, each row represents a spot and each column represents a chromosomal location. Red or blue represent chromosomal gain or loss, respectively.

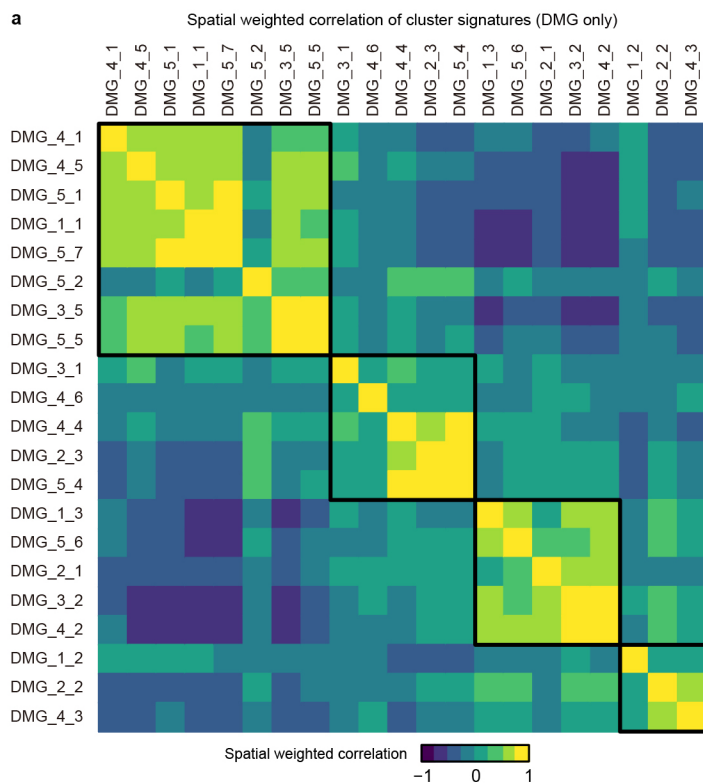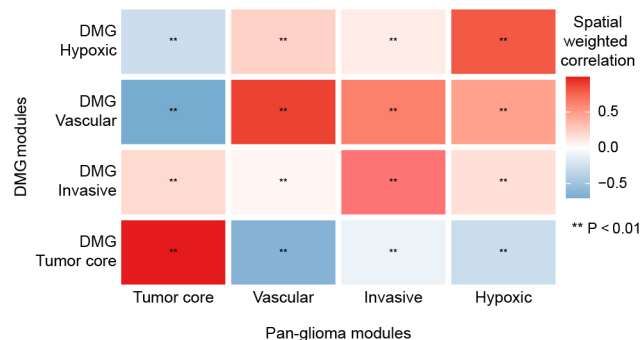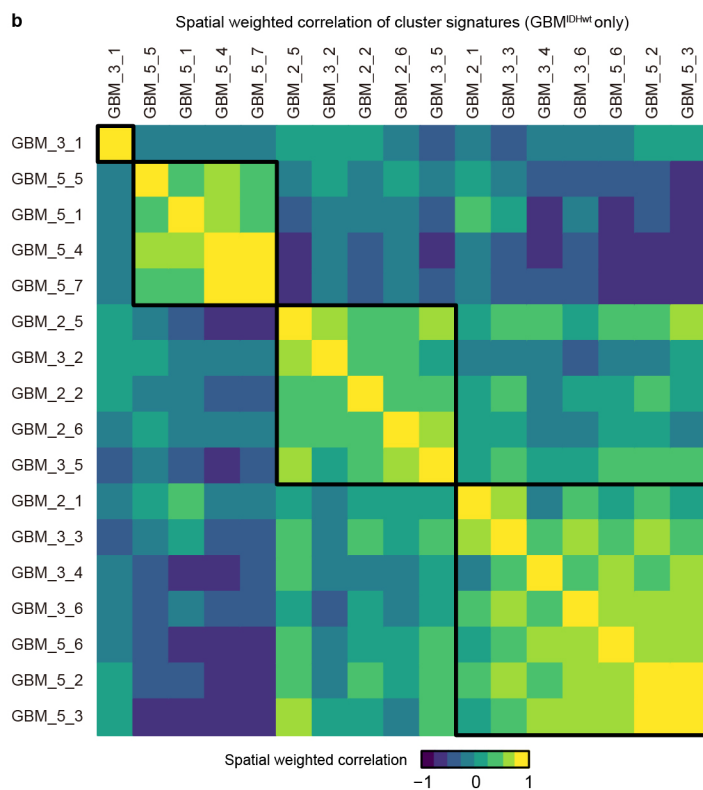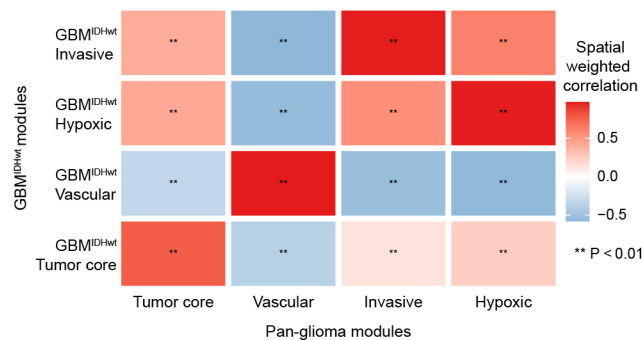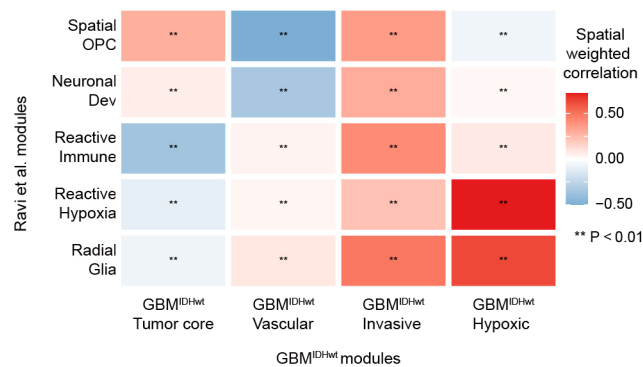

Supplementary Figure 3. Niche-specific spatial transcriptional programs by tumor types.

a) Left: Heatmap of spatially weighted correlation of the 21 spatial cluster signatures from five DMG samples. Hierarchical clustering confirmed 4 clustered programs. Spatial cluster information is marked on the top and left. Right: Heatmap of spatial weighted correlation between DMG modules and pan-glioma modules. Correlation and P values were determined by non-parametric Spearman's correlation test, Bonferroni-corrected for multiple comparisons. Source data are provided as a Source Data file, and individual P values are included.

b) Left: Heatmap of spatially weighted correlation of the 17 spatial cluster signatures from three IDH^{wildtype} GBM samples. Hierarchical clustering confirmed 4 clustered programs. Spatial cluster information is marked on the top and left. Right: Top) Heatmap of spatial weighted correlation between IDH^{wildtype} GBM modules and pan-glioma modules. Bottom) Heatmap of spatial weighted correlation between IDH^{wildtype} GBM modules and Ravi et al. modules. Correlation and P values were determined by non-parametric Spearman's correlation test, Bonferroni-corrected for multiple comparisons. Source data are provided as a Source Data file, and individual P values are included.

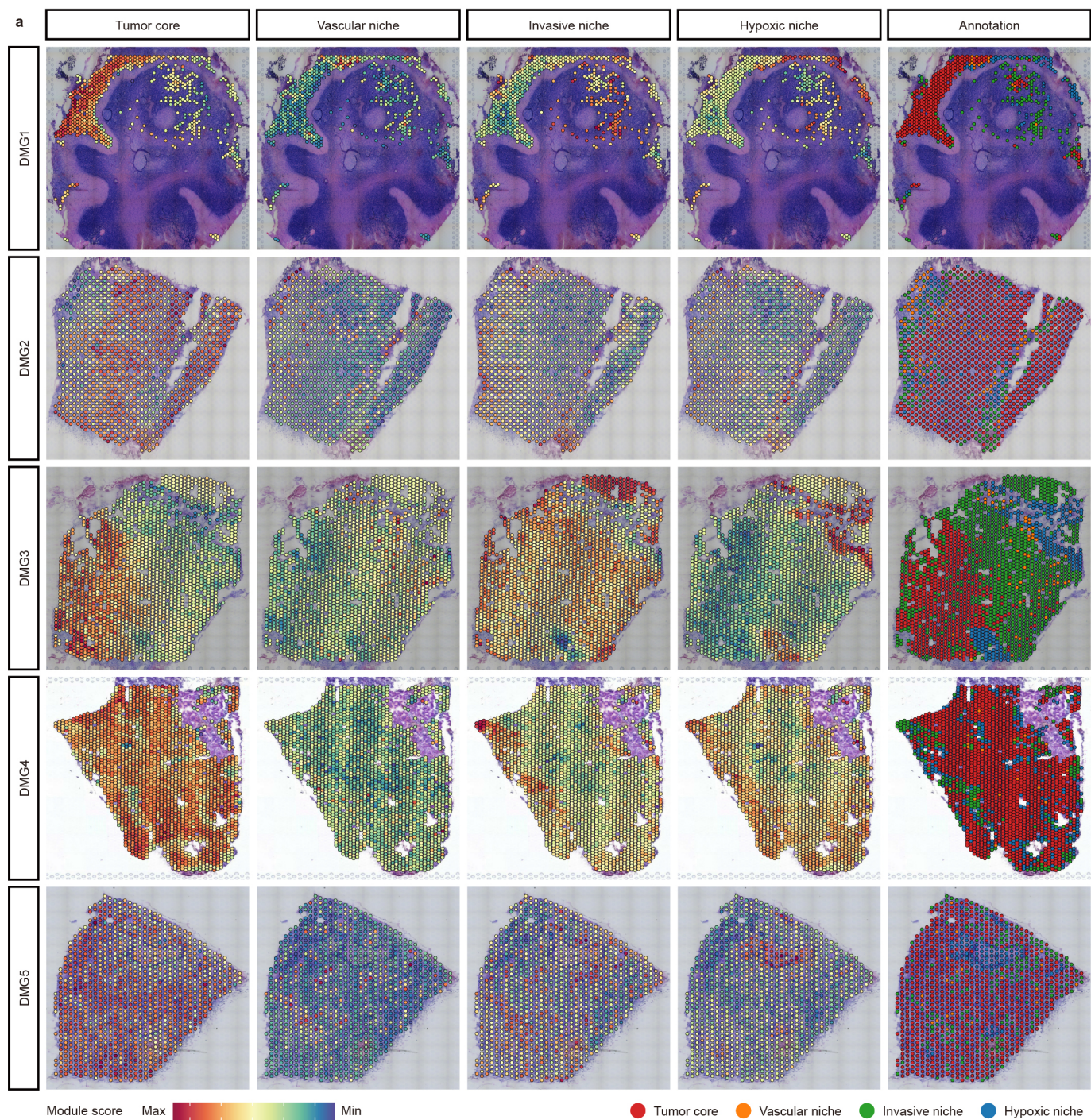

b **DMG3**

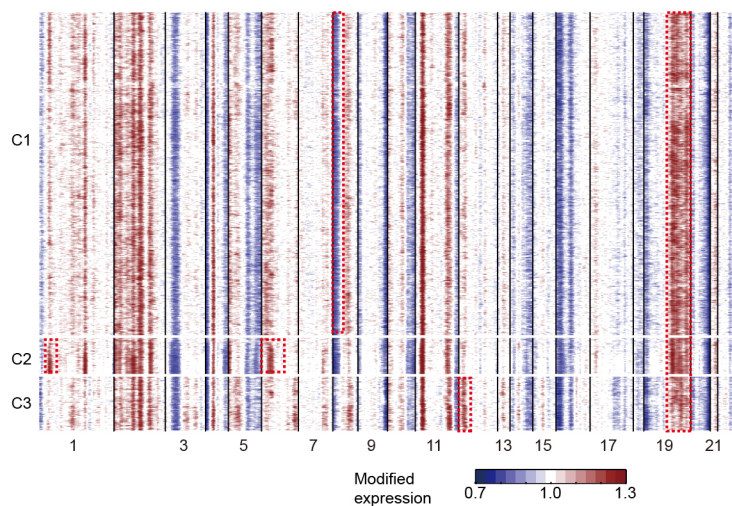

c

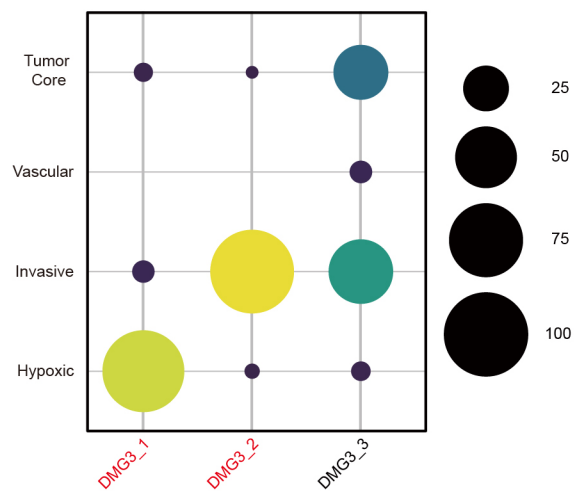

Supplementary Figure 4. Niche-enriched spatial transcriptomic signatures and tumor subclones in DMG samples.

a) Left: scaled expression of Module scores 1-4 corresponding to niche identities in DMG1-5. Right: Each spot is assigned a niche identity based on their highest expressing module score, marked by different colors.

b) An example of subclone assignment based on prominent CNV events (dashed red boxes) in the InferCNV heatmap, showing 3 major subclones (C1-C3) from DMG3.

c) Dot plot indicating the spot distribution of subclones (columns) from DMG3 across different niches (rows). Dot size and color reflect the percentage. The niche-dominant subclones ($\geq 75\%$ in one niche) are highlighted in red.

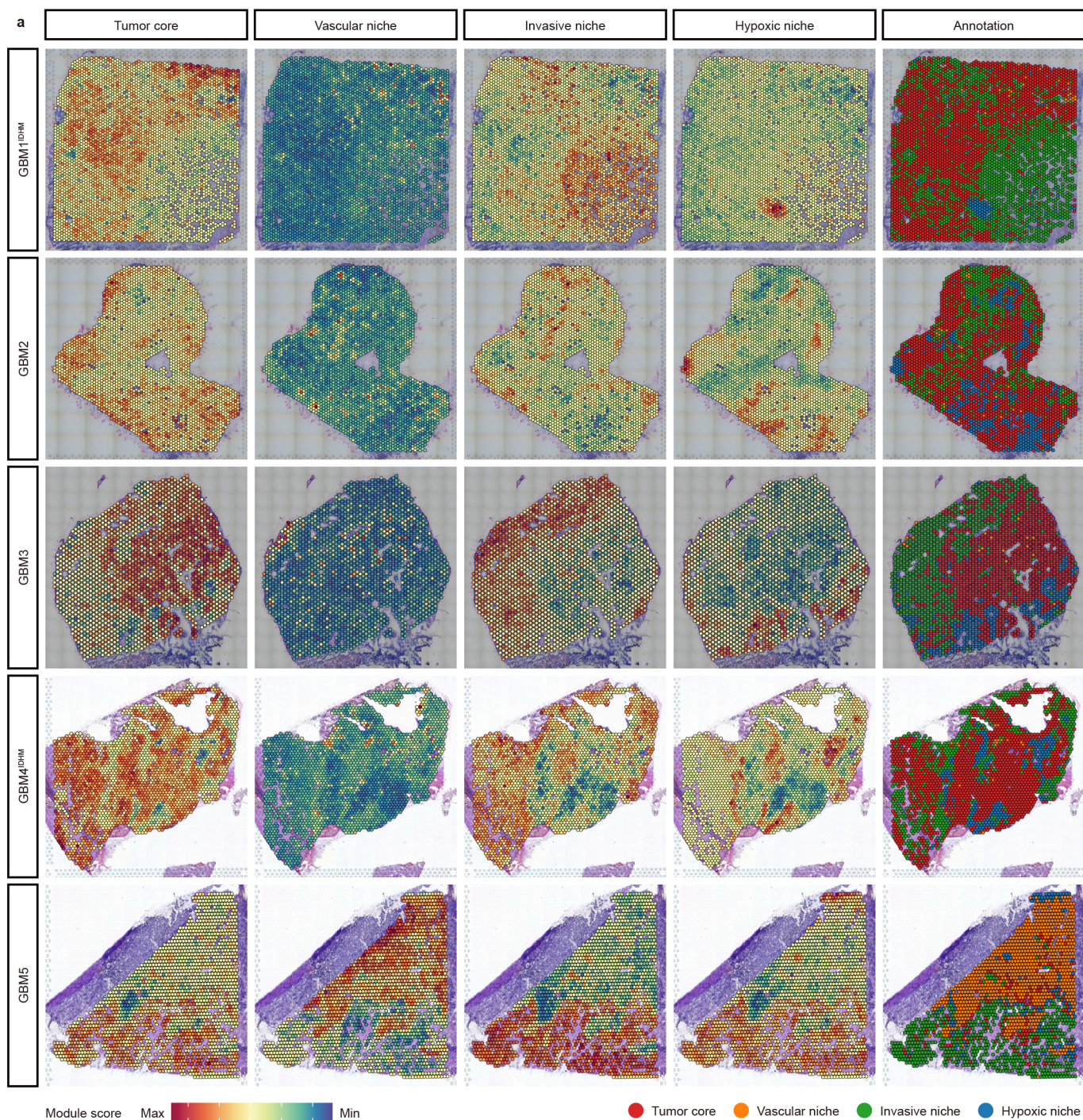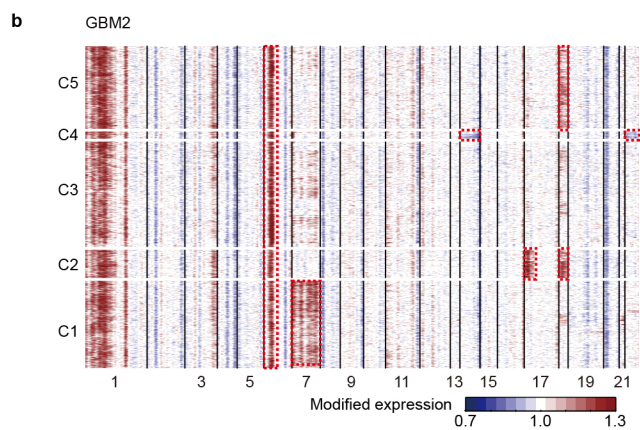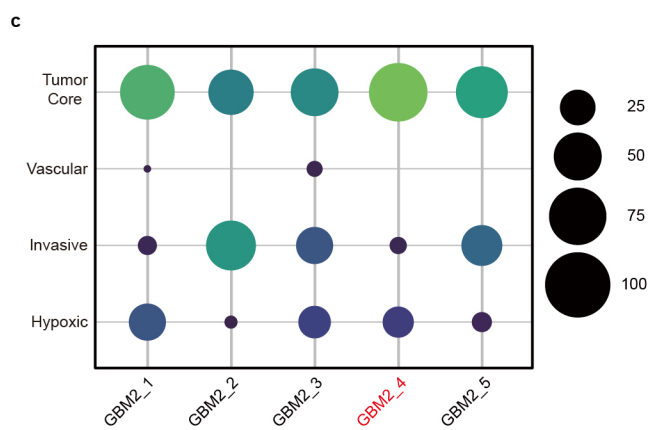

Supplementary Figure 5. Niche-enriched spatial transcriptomic signatures and tumor subclones in DMG samples.

a) Left: scaled expression of Module scores 1-4 corresponding to niche identities in GBM1-5. Right: Each spot is assigned a niche identity based on their highest expressing module score, marked by different colors.

b) An example of subclone assignment based on prominent CNV events (dashed red boxes) in the InferCNV heatmap, showing 5 major subclones (C1-C5) from GBM2.

c) Dot plot indicating the spot distribution of subclones (columns) from DMG3 across different niches (rows). Dot size and color reflect the percentage. The niche-dominant subclone ($\geq 75\%$ in one niche) is highlighted in red.

a Malignant spot distribution

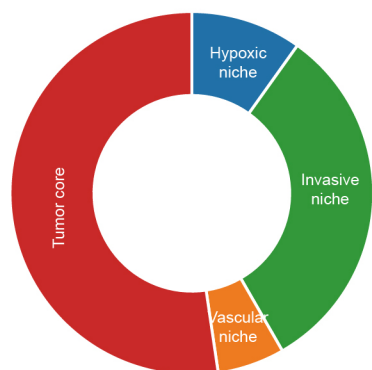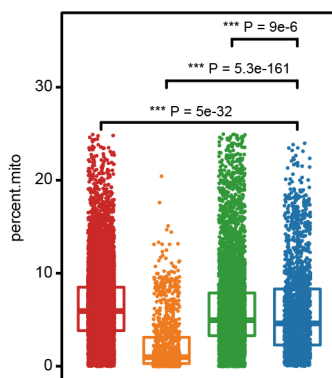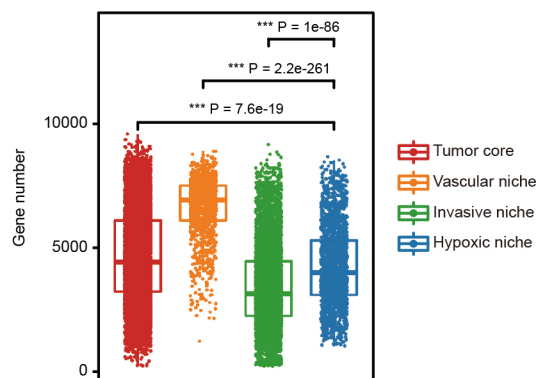

b GBM4

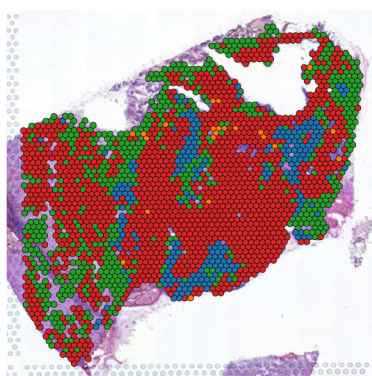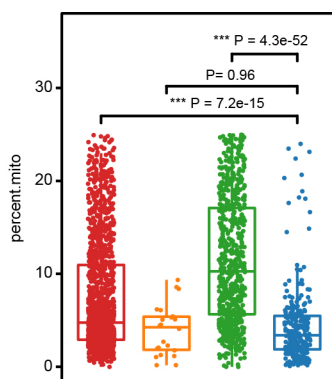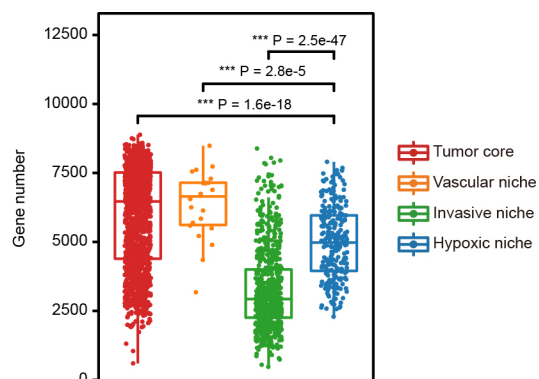

c DMG5

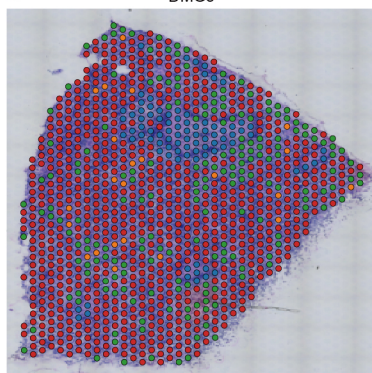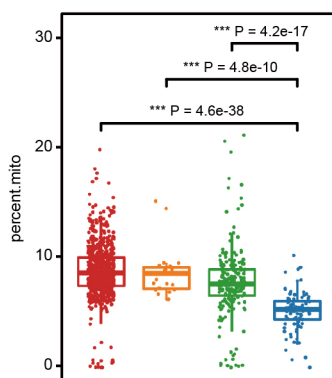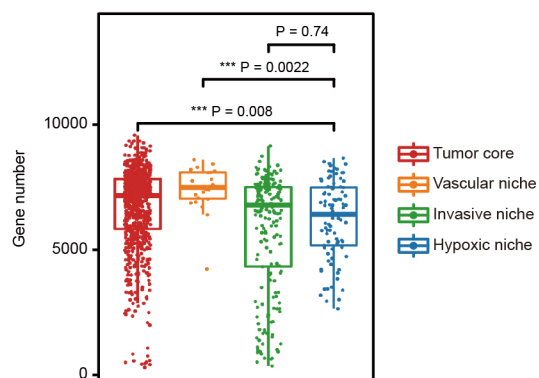

Supplementary Figure 6. Quality control of malignant spots in different niches.

a) Left: Pie chart indicating the percentage of spots assigned to different niches. Right: Box plots of the percentage of mitochondrial genes and the total number of genes in each spot, grouped by niche identity ($n = 10342, 1182, 6290, \text{ and } 1951$ spots, respectively). Boxes indicate quartiles, horizontal bar indicate median, and whiskers indicate range, up to 1.5-fold inter-quartile range. P values, Kruskal–Wallis test for differences between groups with Holm’s correction for multiple comparisons.

b, c) Two examples of samples with prominent pseudopalisading histopathology. Left: spatial plots of four niches for GBM4 (b) and DMG5 (c), distinguished by color. Right: Box plots of the percentage of mitochondrial genes and the total number of genes in each spot from GBM4 ($n = 1447, 22, 645, \text{ and } 249$ spots, respectively) and DMG5 ($n = 750, 23, 194, \text{ and } 89$ spots, respectively), grouped by niche identity. Boxes indicate quartiles, horizontal bar indicate median, and whiskers indicate range, up to 1.5-fold inter-quartile range. P values, Kruskal–Wallis test for differences between groups with Holm’s correction for multiple comparisons.

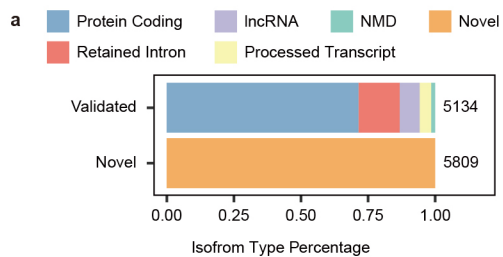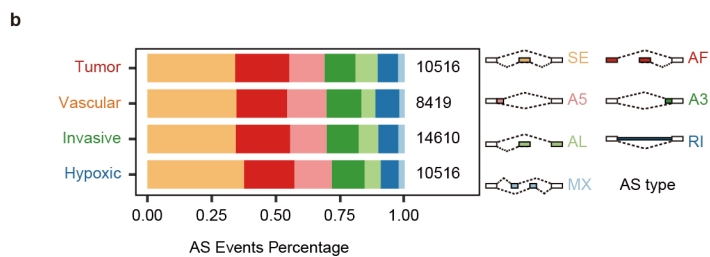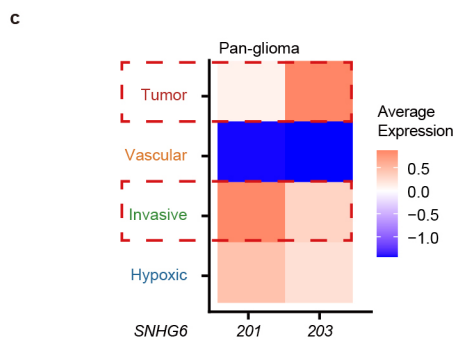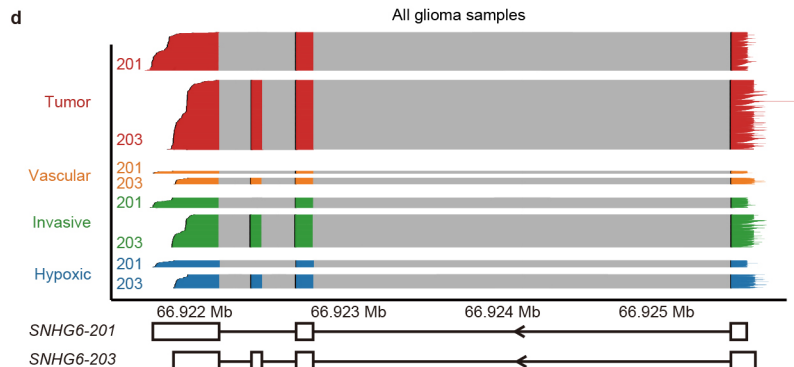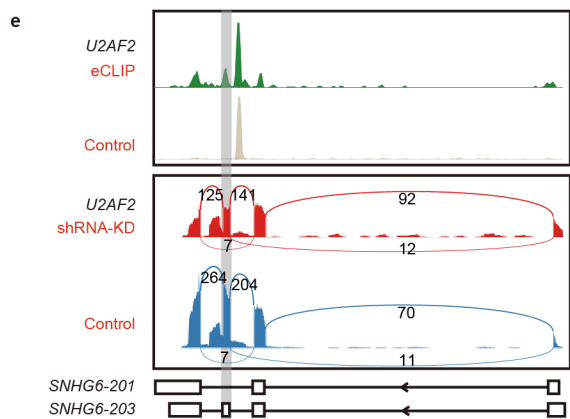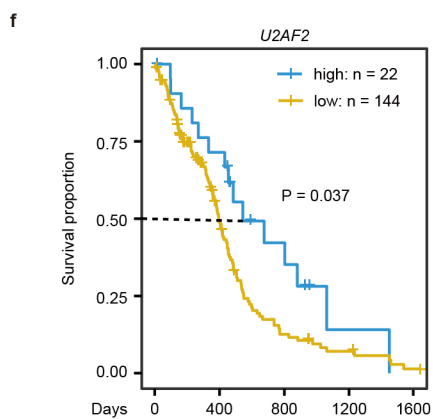

Supplementary Figure 7. RNA isoform diversity across glioma niches.

a) Bar plot of the percentages of isoform types. The total numbers of validated and novel isoforms are labeled. NMD, nonsense-mediated decay.

b) Bar plot of the percentages of AS types in different niches. The total numbers of AS events in each niche are labeled. SE, skipping exon. AF, alternative first exon. A5, alternative 5' splice-site. A3, alternative 3' splice-site. AL, alternative last exon. RI, retained intron. MX, mutually exclusive exons.

c, d) Differential isoform enrichment of *SNHG6-201* and *SNHG6-203* in different niches of all glioma samples (n= 19,071 spots), shown by tile plots (c) and individual transcripts in each niche (d).

e) Predicted splicing regulation of *SNHG6* by splicing factor *U2AF2*. Peaks indicate eCLIP and shRNA-KD RNA-seq read density in HepG2 cell line from ENCODE³⁷. Biological replicates have similar results. Grey box shows the alternatively spliced region of *SNHG6-203*.

f) Kaplan–Meier survival curves comparing the overall survival between *U2AF2* high (n = 12) versus low groups (n = 144) in the TCGA GBM cohort³⁶. Curve comparison P value was determined by two tailed Log-rank (Mantel-Cox) test.

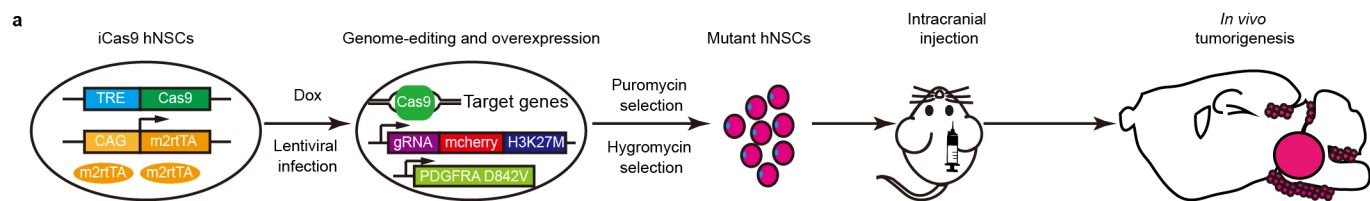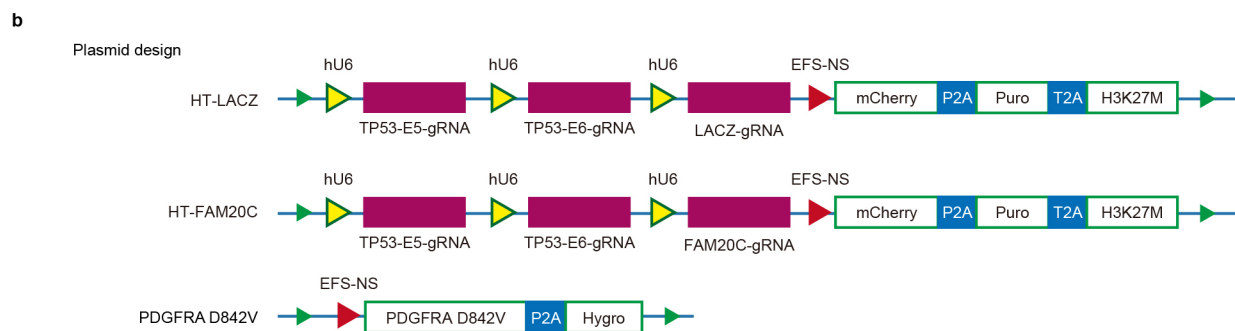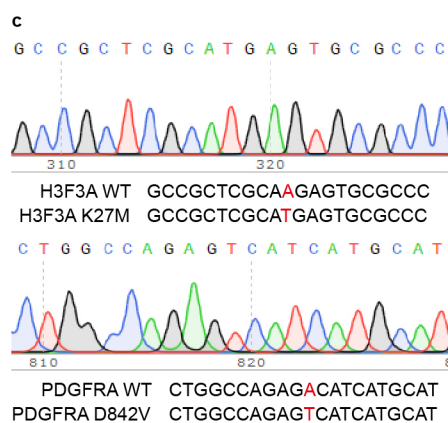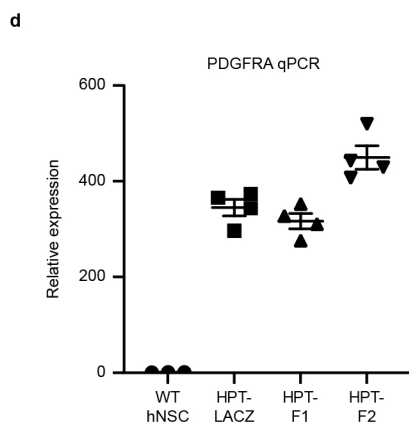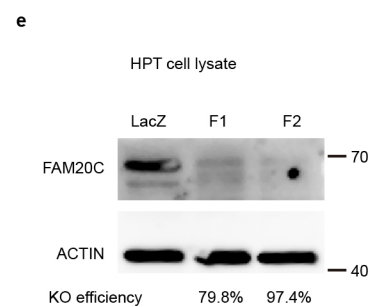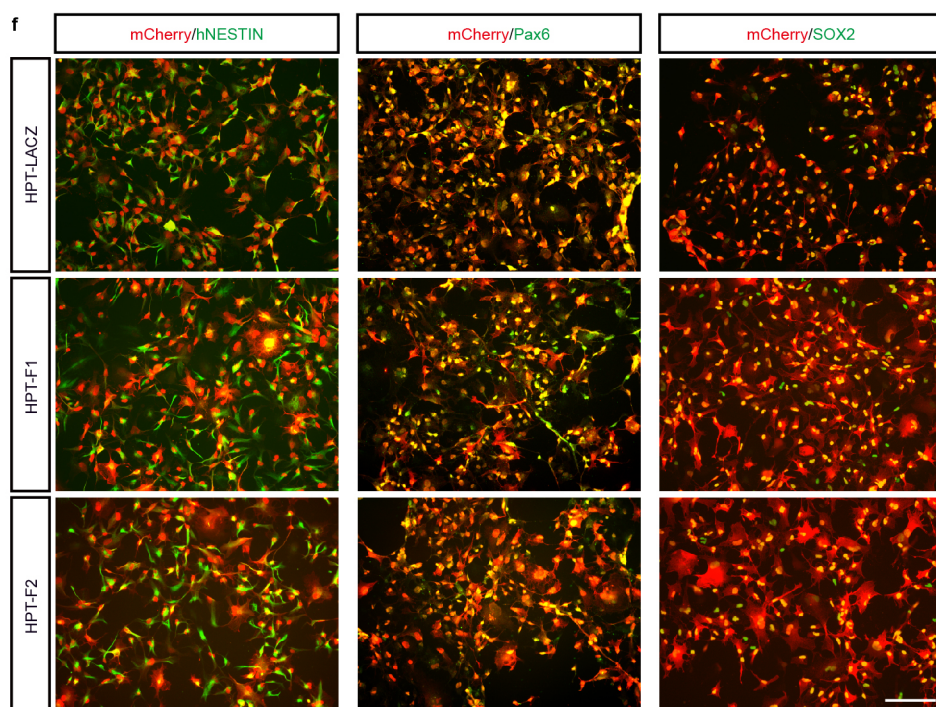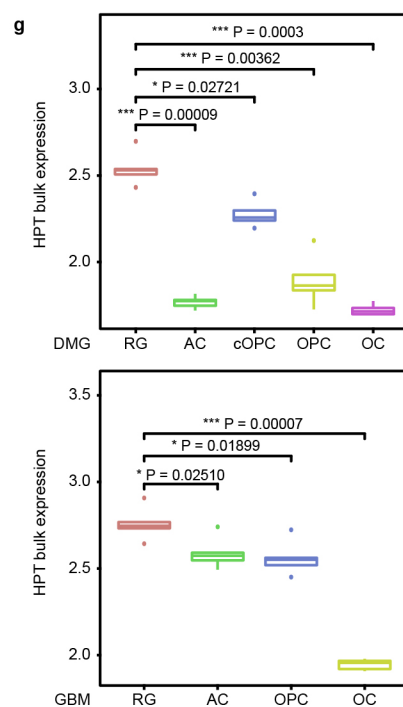

Supplementary Figure 8. Establishment and characterization of DMG-initiating HPT NSCs.

- a) Workflow to generate DMG models from iCas9 hNSCs.
- b) Design of HT-LACZ, HT-FAM20C, and PDGFRA D842V plasmids. sgRNAs targeting *TP53*, *LacZ*, *FAM20C* are driven by independent hU6 promoters. Overexpression of *H3K27M* and *PDGFRA D842V* is driven by EFs-NS promoters. mCherry is used as a reporter. Puromycin (Puro) and Hygromycin (Hygro) resistant transgenes are used for screening. P2A sequences encode self-cleave peptides to separate translated proteins encoded by different transgenes.
- c) Sanger sequencing for *H3K27* and *PDGFRA D842* locus in the constructed plasmids. Mutations are highlighted in red.
- d) qPCR quantifying *PDGFRA* expression in WT, HPT-LACZ, HPT-F1, and HPT-F2 hNSCs. The qPCR experiment was repeated three times. Source data are provided as a Source Data file.
- e) Western Blot analysis for FAM20C protein expression in HPT-LACZ, HPT_F1, and HPT-F2 hNSCs. β -ACTIN were used as loading controls. The knockout efficiency in HPT_F1 and HPT-F2 versus HPT-LACZ is calculated by comparing FAM20C band intensity normalized by β -ACTIN band intensity. The WB experiment was repeated three times. Source data are provided as a Source Data file.
- f) Representative images of mCherry⁺ HPT-LACZ, F1, or F2 cells (n = 3 for each group) stained with hNESTIN, PAX6, and SOX2. Scale bar, 200 μ m.
- g) The relative expression of cell-type specific gene sets (derived from DMG or GBM scRNAseq) in HPT-LACZ hNSCs were compared, based on bulk RNA-seq of HPT-LACZ hNSCs (n = 5). Boxes indicate quartiles, horizontal bar indicate median, and whiskers indicate range, up to 1.5-fold inter-quartile range. P values, Kruskal–Wallis test for between group differences with Benjamini–Hochberg’s correction for multiple comparisons.

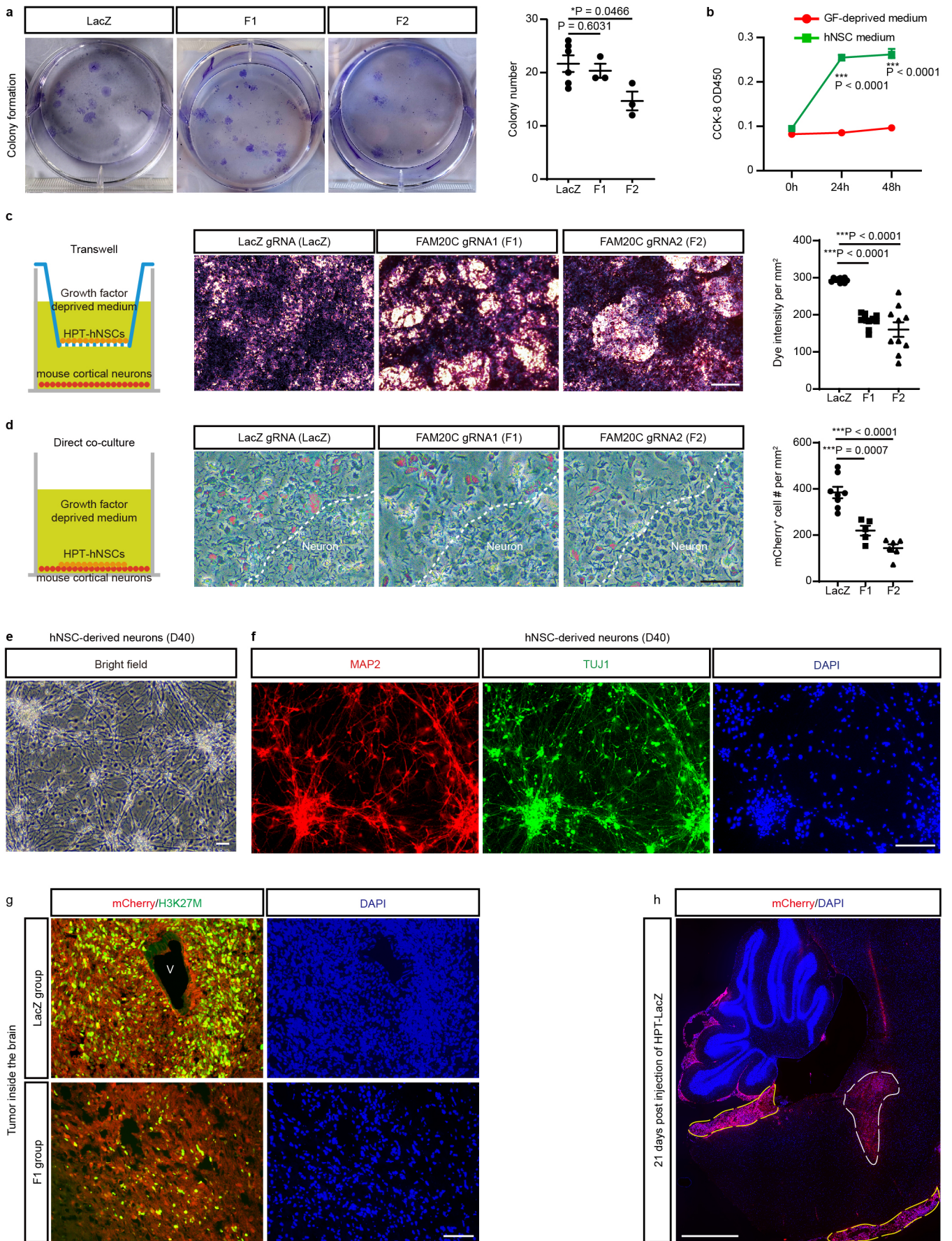

Supplementary Figure 9. FAM20C promotes invasive growth of RG-like cells towards neurons.

a) Left: Representative images of colony formation assay for LacZ (n = 6), F1 (n = 3), and F2 (n = 3) HPT-hNSCs. Right: quantification for the total number of colonies. Scale bar, 1 cm. P values were determined by Student t-test. Error bars indicate mean $\pm$ SEM.

b) Cell proliferation was measured by CCK-8 assay. The growth curves of HPT-hNSCs cultured in normal or growth factor deprived hNSC culture medium from 0h to 48h are compared (n = 3 for each group at different time points). P values were determined by Student t-test. Error bars indicate mean $\pm$ SEM.

c) Left: Illustration of transwell assay with HPT-hNSCs in the top well and mouse cortical neurons in the bottom well in growth factor deprived medium. Middle: Representative images of crest violet staining for migrated LacZ, F1, and F2 HPT-hNSCs (n = 10 for each group). Right: Quantification of dye intensity per mm² membrane for each group. P values were determined by Student t-test. Error bars indicate mean $\pm$ SEM. Scale bar, 200 μ m.

d) Left: Illustration of direct co-culture of HPT-hNSCs with mouse cortical neurons in growth factor deprived medium. Middle: Representative images of mCherry⁺ LacZ (n = 8), F1 (n = 5), and F2 (n = 6) HPT-hNSCs adjacent to high-density neuronal clusters. Right: Quantification of the density of mCherry⁺ HPT-hNSCs adjacent to high-density neuronal clusters for each group. P values were determined by Student t-test. Error bars indicate mean $\pm$ SEM. Scale bar, 200 μ m.

e, f) Representative images of neurons differentiated from hNSCs at Day 40, bright field (e) (n = 3) or immunostained for MAP2/TUJ1/DAPI (f) (n = 3). Scale bars, 50 μ m in e and 200 μ m in f.

g) Representative images of IF co-labeling of mCherry/H3K27M for tumor areas inside the brains of LacZ and F1 groups (n = 10 for each group). V, blood vessel. Scale bar, 50 μ m.

h) A representative image showing the distribution of mCherry⁺ cells in the hindbrain 21 days post injection of HPT-LacZ hNSCs (n = 4). Scale bar, 1 mm.

Source data for a-d are provided as a Source Data file.
