# Supplementary material for: Spatial transcriptomics reveals niche-specific enrichment and vulnerabilities of radial glial stem-like cells in malignant gliomas
Source: Nat Commun. 2023 Feb 23;14:1028. doi: 10.1038/s41467-023-36707-6 (PMC9950149; doi:10.1038/s41467-023-36707-6)
Supplement: Supplementary file 3 — Description of Additional Supplementary Files [file 41467_2023_36707_MOESM3_ESM.pdf]

**Description of Additional Supplementary Files**

File Name: Supplementary  Data 1
Description: Patient sample information.

File Name: Supplementary Data 2
Description: Quality control for short- and long-read transcriptomes.

File Name: Supplementary Data 3
Description: Four integrated gene sets of spatial transcriptional modules.

File Name: Supplementary Data 4
Description: Gene Ontology enrichment analysis of Biological Process (BP) for all glioma modules.

File Name: Supplementary  Data 5
Description: Niche-specific differentially expressed isoforms across all samples.

File Name: Supplementary Data 6
Description: Survival-related splice junctions from differentially expressed isoforms.

File Name: Supplementary Data 7
Description: A list of RG-Vs-AC-specific genes based on Bhaduri et al. GBM scRNA-seq.

File Name: Supplementary Data 8
Description: Predicted ligand-receptor pairs in each module.

File Name: Supplementary Data 9
Description: Gene expression dynamics along the spatial tumor invasion trajectory.

File Name: Supplementary Data 10
Description: Gene Ontology enrichment analysis of BP for genes in the GCL_TI_RG signature.
