# Supplementary material for: Spatial transcriptomics reveals niche-specific enrichment and vulnerabilities of radial glial stem-like cells in malignant gliomas
Source: Nat Commun. 2023 Feb 23;14:1028. doi: 10.1038/s41467-023-36707-6 (PMC9950149; doi:10.1038/s41467-023-36707-6)
Supplement: Supplementary file 14 — Reporting Summary [file 41467_2023_36707_MOESM14_ESM.pdf]

nature portfolio

Corresponding author(s): Yuan Wang
Last updated by author(s): Feb 3, 2023

Reporting Summary

Nature Portfolio wishes to improve the reproducibility of the work that we publish. This form provides structure for consistency and transparency in reporting. For further information on Nature Portfolio policies, see our Editorial Policies and the Editorial Policy Checklist.

Statistics

For all statistical analyses, confirm that the following items are present in the figure legend, table legend, main text, or Methods section.

n/a	Confirmed	
☐	☒	The exact sample size (*n*) for each experimental group/condition, given as a discrete number and unit of measurement
☐	☒	A statement on whether measurements were taken from distinct samples or whether the same sample was measured repeatedly
☐	☒	The statistical test(s) used AND whether they are one- or two-sided<br>*Only common tests should be described solely by name; describe more complex techniques in the Methods section.*
☒	☐	A description of all covariates tested
☐	☒	A description of any assumptions or corrections, such as tests of normality and adjustment for multiple comparisons
☐	☒	A full description of the statistical parameters including central tendency (e.g. means) or other basic estimates (e.g. regression coefficient) AND variation (e.g. standard deviation) or associated estimates of uncertainty (e.g. confidence intervals)
☐	☒	For null hypothesis testing, the test statistic (e.g. *F*, *t*, *r*) with confidence intervals, effect sizes, degrees of freedom and *P* value noted<br>*Give P values as exact values whenever suitable.*
☒	☐	For Bayesian analysis, information on the choice of priors and Markov chain Monte Carlo settings
☒	☐	For hierarchical and complex designs, identification of the appropriate level for tests and full reporting of outcomes
☐	☒	Estimates of effect sizes (e.g. Cohen's *d*, Pearson's *r*), indicating how they were calculated

Our web collection on statistics for biologists contains articles on many of the points above.

Software and code

Policy information about availability of computer code

Data collection	All software used for data collection is commercially available and stated in the Methods section. Illumina NovaSeq platform was used for spatial transcriptomic sequencing. Real-time quantitative PCR (RT-qPCR) was performed in BioRad CFX96 Touch Real-Time PCR Detection System. Immunofluorescent staining for tissue slides were imaged under a Olympus BX51 fluorescent microscope. Immunofluorescent staining for cells were imaged under a Zeiss (LSM880) confocal microscope.
Data analysis	The following packages were used for processing and data analysis of spatial transcriptomic sequencing data: R (v4.0.3), SpaceRanger (v1.1.0), SpotClean (v0.99.2), Guppy (v5.0.11), minimap (v2.22), ScNapBar (v1.0.0), pychopper (v2.5.0), TranscriptClean (v2.0.2), InferCNV (v1.7.1), REDItools (v2), Seurat (v4.0.4), SpatialPCA (v1.2.0), Spruce (v0.99.1), spatialDE2 (v2), Banksy (v0.1.3), corrplot (v0.92), GWmodel (v2.2-9), gwrr (v0.2-2), ComplexHeatmap (v2.0.0), clusterProfiler (v4.6.0), RCTD (v1.1.0), StringTie2 (v2.1.7), SUPPA2 (v2.3), GenomicRanges (v1.40.0), Samtools (v1.9), Bedtools (v2.29.2), GSVA (v1.46.0), ggbio (v1.38.0), survminer (v0.4.9), CellChat (v1.4.0), Nichenetr (v1.0.0) , SPATA2 (v0.1.0), ggplot2 (v3.3.6)<br><br>Plotting and statistical analysis was performed in the R statistical environment (v4.0.3) and GraphPad Prism (v9).

For manuscripts utilizing custom algorithms or software that are central to the research but not yet described in published literature, software must be made available to editors and reviewers. We strongly encourage code deposition in a community repository (e.g. GitHub). See the Nature Portfolio guidelines for submitting code & software for further information.

Data

Policy information about availability of data

All manuscripts must include a data availability statement. This statement should provide the following information, where applicable:

- Accession codes, unique identifiers, or web links for publicly available datasets
- A description of any restrictions on data availability
- For clinical datasets or third party data, please ensure that the statement adheres to our policy

The raw data for short-read sequencing in this study were deposited in Genome Sequence Archive with accession ID HRA001865 (https://ngdc.cncb.ac.cn/gsa-human/browse/HRA001865). The raw data for long-read sequencing in this study were deposited in Genome Sequence Archive with accession ID HRA001960 (https://ngdc.cncb.ac.cn/gsa-human/browse/HRA001960). The raw data for bulk RNA-seq of HPT cells were deposited in Genome Sequence Archive with accession ID HRA003511 (https://ngdc.cncb.ac.cn/gsa-human/browse/HRA003511).

Since these data are related to human genetic resources, raw data can be obtained within 3-6 weeks by requesting and following the guidelines for Genome Sequence Archive for noncommercial use. There are no time restrictions once access has been granted. The guidance for making a data access request of GSA for humans can be downloaded at the National Genomics Data Center website (https://ngdc.cncb.ac.cn/gsa-human/document/GSA-Human_Request_Guide_for_Users_us.pdf). The processed data in this study have been deposited in the GEO database under the accession GSE194329 (https://www.ncbi.nlm.nih.gov/gds/?term=GSE194329) and Figshare (https://doi.org/10.6084/m9.figshare.20653908). The corresponding author will respond to requests for the data within one week.

The TCGA GBM publicly available data used in this study are available in the TCGA Data Portal (https://tcga-data.nci.nih.gov/tcga/). The Bhaduri et al. GBM scRNA-seq publicly available data used in this study are available in UCSC Cell Browser (http://gbm.cells.ucsc.edu). The Filbin et al. DIPG scRNA-seq publicly available data used in this study are available in Broad Single Cell Portal (https://singlecell.broadinstitute.org/single_cell). The eCLIP and HepG2 shRNA knockdown publicly available data used in this study are available in the ENCODE Data portal (https://www.encodeproject.org). The Nowakowski et al. human cortex scRNA-seq publicly available data used in this study are available in UCSC Cell Browser ((http://cells.ucsc.edu/?ds=cortex-dev). The Aldinger et al. human cerebellum snRNA-seq publicly available data used in this study are available in Human Cell Atlas (https://www.covid19cellatlas.org/aldinger20) and the UCSC Cell Browser (https://cbl-dev.cells.ucsc.edu). The remaining data are available within the Article, Supplementary Information or Source Data file. Source data are provided with this paper.

Human research participants

Policy information about studies involving human research participants and Sex and Gender in Research.

Reporting on sex and gender	Sex or gender was not considered in this study, since it does not have a major impact on the spatial transcriptomes of gliomas.
Population characteristics	Detailed patient information is listed in Supplementary Data 1.
Recruitment	Patients requiring surgical resection of GBM and DMG who provided written consent were recruited at West China Hospital from 2020.09 to 2021.03. No self-selection bias or other biases is present.
Ethics oversight	This study was approved by the Ethics Committee on Biomedical Research of West China Hospital, Sichuan University, Chengdu, China (Approval number: 2020.837).

Note that full information on the approval of the study protocol must also be provided in the manuscript.

Field-specific reporting

Please select the one below that is the best fit for your research. If you are not sure, read the appropriate sections before making your selection.

☒ Life sciences ☐ Behavioural & social sciences ☐ Ecological, evolutionary & environmental sciences

For a reference copy of the document with all sections, see nature.com/documents/nr-reporting-summary-flat.pdf

Life sciences study design

All studies must disclose on these points even when the disclosure is negative.

Sample size	No sample size calculation was performed. We followed the routine biological replicate requirement in the field, n > = 3 for each group.
Data exclusions	We excluded spatial transcriptomes following a pre-established criteria (gene count < 200 or the mitochondria gene ratio > 25%). Transciptomes that failed this criteria are considered low-quality, often resulting from RNA degradation or poor library preparation.
Replication	We confirm that all attempts at replication were successful. For each experiment, we performed at least two repeats.
Randomization	Samples and mice were always randomly assigned to each group.

Blinding

For in vitro and in vivo experiments, data collection was performed by one researcher, and the quantification were done by another researcher in a blind way.

Reporting for specific materials, systems and methods

We require information from authors about some types of materials, experimental systems and methods used in many studies. Here, indicate whether each material, system or method listed is relevant to your study. If you are not sure if a list item applies to your research, read the appropriate section before selecting a response.

Materials & experimental systems

n/a	Involved in the study
☐	☒ Antibodies
☐	☒ Eukaryotic cell lines
☒	☐ Palaeontology and archaeology
☐	☒ Animals and other organisms
☒	☐ Clinical data
☒	☐ Dual use research of concern

Methods

n/a	Involved in the study
☒	☐ ChIP-seq
☒	☐ Flow cytometry
☐	☒ MRI-based neuroimaging

Antibodies

Antibodies used

Primary antibodies:
anti-β-actin (1:1000, rabbit, Cell Signaling Technology, 93473SF)
anti-FAM20C (1:500, rabbit, Abcam, ab154740)
anti-OLIG2 (1:1000, rabbit, Abcam, AB9610)
anti-KI67 (1:500, rabbit, BD, 550609)
anti-mCherry (1:1000, chicken, Abcam, ab205402)
anti-Human Nuclear Antigen antibody (1:200, mouse, Abcam, ab191181)
anti-MAP2 (1:5000, Chicken, Novus, NB300-213)
anti-TUJ1 (1:5000, Rabbit, Sigma, 801201)
anti-hNESTIN (1:1000, mouse, abcam, ab22035)
anti-Sox2 (1:1000, Rabbit, Abcam, ab92494)
anti-Pax6 (1:1000, Rabbit, abcam, ab5790)

Secondary antibodies:
anti-Rabbit IgG (H+L) Antibody, Peroxidase-Labeled (1:10000, SeraCare, 5220-0336)
Goat anti-Chicken IgY (H+L) Secondary Antibody, Alexa Fluor™ 555 (1:1000, Invitrogen, A-21437)
Goat anti-Mouse IgG (H+L) Cross-Adsorbed Secondary Antibody, Alexa Fluor™ 488 (1: 1000, Invitrogen, A-11001)
Goat anti-Rabbit IgG (H+L) Cross-Adsorbed Secondary Antibody, Alexa Fluor™ 488 (1: 1000, Invitrogen, A-11008)
Goat anti-Mouse IgG (H+L) Cross-Adsorbed Secondary Antibody, Alexa Fluor™ 647 (1: 1000, Invitrogen, A-21235)

Validation

All the antibodies have been manufacturer-validated for the use of immunohistochemistry (IHC), immunofluorescence (IF), and/or WB analyses, briefly summarized below. Detailed descriptions are available on the manufacturer's website.

β-actin (validated for WB)
FAM20C (validated for WB, IHC-P, ICC/I)
OLIG2 (validated for IC, IHC, IP, WB)
Ki67 (validated for IHC-Fr)
mCherry (validated for WB, ICC/IF. Positive control: Lysate of HEK293 cells transfected with pFin-EF1-mCherry vector; HEK293 cells transfected with pFin-EF1-mCherry vector)
Human Nuclear Antigen antibody (validated for Flow Cyt, ICC/IF, Positive control: IHC-Fr: Human tonsil tissue; ICC: MCF and K562 cells; Flow Cyt: Raji, MCF7, K562, HeLa, and Jurkat cells)
MAP2 (validated for WB, IHC/IF, knockdown validated)
TUJ1 (validated for IHC-P)
hNESTIN (validated for ICC, WB. Positive control, WB: U251 cells. ICC: U251 cells. Human brain tissue)
Sox2 (validated for WB, IHC-P, ICC/IF. Positive control, ICC/IF: F9 and NCCIT cells; mouse neuromesodermal progenitors)
Pax6 (validated for IP, ICC/IF, WB. Positive control, ICC: ATRA treated NCCIT cells; WB: rat whole eye extract; human 293T, HepG2, U20S, MCF7, Jurkat NCCIT cells; monkey COS7, mouse C2C12 cells; IP: 293T cells)

Eukaryotic cell lines

Policy information about cell lines and Sex and Gender in Research

Cell line source(s)

The iCas9 hPSCs were gifted by Dr. Danwei Huangfu at Sloan-Kettering Institute; HEK-293T was purchased from National Collection of Authenticated Cell Cultures (Shanghai, China) (Catalog number GNhu177).

Authentication

All cell lines were authenticated by the supplier. We performed additional cell line authentication by STR profiling on both iCas9 hPSCs and HEK-293T cells (TsingKe Biological Technology, Beijing, China), and confirmed their identities.

March 2021

Mycoplasma contamination	Cell lines were routinely checked for mycoplasma contamination. All cell lines used in this study were tested negative for mycoplasma.
Commonly misidentified lines (See ICLAC register)	No commonly misidentified cell lines were used in the study.

Animals and other research organisms

Policy information about studies involving animals; ARRIVE guidelines recommended for reporting animal research, and Sex and Gender in Research

Laboratory animals	4–5 weeks old female NCG mice from GemPharmatech, Ltd., Nanjing, China.
Wild animals	The study did not involve wild animals.
Reporting on sex	Sex was not considered in this study, since it does not have a major impact on animal model development or xenograft growth. Female mice were used because they are easier to handle and pool than male mice.
Field-collected samples	The study did not involve field-collected samples.
Ethics oversight	All animal studies were approved by the Animal Care and Use Committee of Sichuan University.

Note that full information on the approval of the study protocol must also be provided in the manuscript.

Magnetic resonance imaging

Experimental design

Design type	MRI to evaluate the brain tumor size and location for individual recruited patients requiring surgical resection of GBM or DMG .
Design specifications	MRI was performed on individual recruited patients requiring surgical resection of GBM or DMG according to the clinical schedules.
Behavioral performance measures	No behavioral performance was measured.

Acquisition

Imaging type(s)	Postcontrast enhanced T1-weighted MRI images.
Field strength	3T
Sequence & imaging parameters	Standard parameters of the radiology departments of the institutions: repetition time: 1550 ms, echo time: 1.98 ms, slice thickness: 5~6 mm, axial imaging.
Area of acquisition	We used whole brain MRI.

Diffusion MRI ☐ Used ☒ Not used

Preprocessing

Preprocessing software	No preprocessing was used.
Normalization	No normalization was used.
Normalization template	No template was used.
Noise and artifact removal	No noise or artifact removal was used.
Volume censoring	No volume sensing was used.

Statistical modeling & inference

Model type and settings	No statistical modeling was used.
Effect(s) tested	No statistical effects were tested

Specify type of analysis: ☒ Whole brain ☐ ROI-based ☐ Both

Statistic type for inference (See Eklund et al. 2016)	None used.
Correction	None needed.

Models & analysis

n/a	Involved in the study
☒	☐ Functional and/or effective connectivity
☒	☐ Graph analysis
☒	☐ Multivariate modeling or predictive analysis
